# NSAID-Exacerbated Respiratory Disease (NERD): From Pathogenesis to Improved Care

**DOI:** 10.3389/fphar.2020.01147

**Published:** 2020-07-28

**Authors:** Seong-Dae Woo, Quoc Quang Luu, Hae-Sim Park

**Affiliations:** ^1^ Department of Allergy and Clinical Immunology, Ajou University School of Medicine, Suwon, South Korea; ^2^ Department of Biomedical Sciences, Ajou University School of Medicine, Suwon, South Korea

**Keywords:** nonsteroidal antiinflammatory drugs, hypersensitivity, asthma, rhinitis, eosinophil, leukotrienes, diagnosis, treatment

## Abstract

Nonsteroidal antiinflammatory drug (NSAID)-exacerbated respiratory disease (NERD) is characterized by moderate-to-severe asthma and a higher prevalence of chronic rhinosinusitis/nasal polyps, but is a highly heterogeneous disorder with various clinical manifestations. Two major pathogenic mechanisms are: (1) overproduction of cysteinyl leukotrienes with dysregulation of arachidonic acid metabolism and (2) increased type 2 eosinophilic inflammation affected by genetic mechanisms. Aspirin challenge is the gold standard to diagnose NERD, whereas reliable *in vitro* biomarkers have yet not been identified. Therapeutic approaches have been done on the basis of disease severity with the avoidance of culprit and cross-reacting NSAIDs, and when indicated, aspirin desensitization is an effective treatment option. Biologic approaches targeting Type 2 cytokines are emerging as potential therapeutic options. Here, we summarize the up-to-date evidence of pathophysiologic mechanisms and diagnosis/management approaches to the patients with NERD with its phenotypic classification.

## Introduction

Aspirin (acetylsalicylic acid, ASA) and nonsteroidal antiinflammatory drugs (NSAIDs) are the most commonly prescribed drugs in the world (Doña et al., 2012); however, they are considered the most common causes of hypersensitivity reactions to drugs (Blanca-Lopez et al., 2018). Hypersensitivity reactions to NSAIDs have recently been classified by the European Academy of Allergy and Clinical Immunology (EAACI) and European Network of Drug Allergy (ENDA): 1) pharmacologic reactions (mediated by cyclooxygenase [COX]-1 inhibitions) include NSAID-exacerbated respiratory disease (NERD), NSAID-exacerbated cutaneous disease (NECD) and NSAID-induced urticarial/angioedema (NIUA), and present cross-intolerance to various COX-1 inhibitors; 2) selective responses (mediated by immunologic mechanisms) include single NSAIDs-induced urticaria, angioedema and/or anaphylaxis (SNIUAA) and single NSAIDs-induced delayed hypersensitivity reactions (SNIDHR) (Kowalski and Stevenson, 2013). NERD is a major phenotype among cross-intolerant categories of NSAID hypersensitivity and had been called ASA-induced asthma, ASA-intolerant asthma, ASA-sensitive asthma; however, NERD and ASA-exacerbated respiratory disease (AERD) are commonly used (Sánchez-Borges, 2019). The prevalence of NERD is reported to be 5.5% to 12.4% in the general population (Lee et al., 2018a; Chu et al., 2019; Taniguchi et al., 2019), 7.1% among adult asthmatics and 14.9% among severe asthmatics (Rajan et al., 2015), while it rarely occurs in children (Taniguchi et al., 2019). No relationships were found with family history or NSAID administration history (Kowalski et al., 2011; Taniguchi et al., 2019).

NERD is characterized by moderate-to-severe asthma and a higher prevalence of chronic rhinosinusitis (CRS) nasal polyps (NPs) with persistent eosinophilic inflammation in the upper and lower airways (Taniguchi et al., 2019) as well as NSAID hypersensitivity where cysteinyl leukotrienes (CysLTs) over-production and chronic type 2 airway inflammation are key findings (Taniguchi et al., 2019). The diagnosis of NERD is confirmed by ASA challenge (*via* orally, bronchially or nasally route) and supported by potential biomarkers (Pham et al., 2017; Cingi and Bayar Muluk, 2020). In addition, *in vitro* cell activation tests and radiological imaging with nasal endoscopy can aid in NERD diagnosis (Taniguchi et al., 2019). This review updates the current knowledge on pathophysiologic mechanisms including molecular genetic mechanisms as well as the diagnosis and treatment of NERD.

## Clinical Features

NERD is characterized by chronic type 2 inflammation in the upper and lower airways; therefore, patients suffer from chronic persistent asthmatic symptoms and CRS with/without NPs, which are exacerbated by ASA/NSAID exposure and refractory to conventional medical or surgical treatment. Some patients are accompanied by cutaneous symptoms such as urticaria, angioedema, flushing or gastrointestinal symptoms (Buchheit and Laidlaw, 2016). Previous studies suggested that NERD is more common in females (middle-age onset) and non-atopics (Choi et al., 2015; Trinh et al., 2018). It was reported that rhinitis symptoms appear and then evolve into CRS which worsens asthmatic symptoms, subsequently followed by ASA intolerance (Szczeklik et al., 2000). However, their clinical presentations and courses have been found to be heterogeneous. It has been increasingly required to classify the subphenotypes of NERD according to its clinical features. One study demonstrated 4 subphenotypes by applying a latent class analysis in a Polish cohort: class 1 patients showing moderate asthma with upper airway symptoms and blood eosinophilia; class 2 patients showing mild asthma with low healthcare use; class 3 patients showing severe asthma with severe exacerbation and airway obstruction; and class 4 patients showing poorly controlled asthma with frequent and severe exacerbation (Bochenek et al., 2014). Another study showed 4 subtypes presenting distinct clinical/biochemical findings in a Korean cohort using a 2-step cluster analysis based on 3 clinical phenotypes (urticaria, CRS and atopy status): subtype 1 (NERD with CRS/atopy and no urticaria), subtype 2 (NERD with CRS and no urticaria/atopy), subtype 3 (NERD without CRS/urticaria), and subtype 4 (NERD with acute/chronic urticaria exacerbated by NSAID exposure) (Lee et al., 2017). Each subtype had distinct features in the aspect of female proportion, the degree of eosinophilia, leukotriene (LT) E_4_ metabolite levels, the frequency of asthma exacerbation, medication requirements (high-dose ICS-LABA or systemic corticosteroids) and asthma severity, suggesting that stratified strategies according to subtype classification may help achieve better clinical outcomes in the management of NERD.

## Pathophysiology

The major upper and lower airway symptoms of NERD are mediated by increased levels of CysLTs with dysregulation of arachidonic acid (AA) metabolism and intense type 2/eosinophilic inflammation (Cingi and Bayar Muluk, 2020).

### CysLTs Overproduction

**Figure 1:**
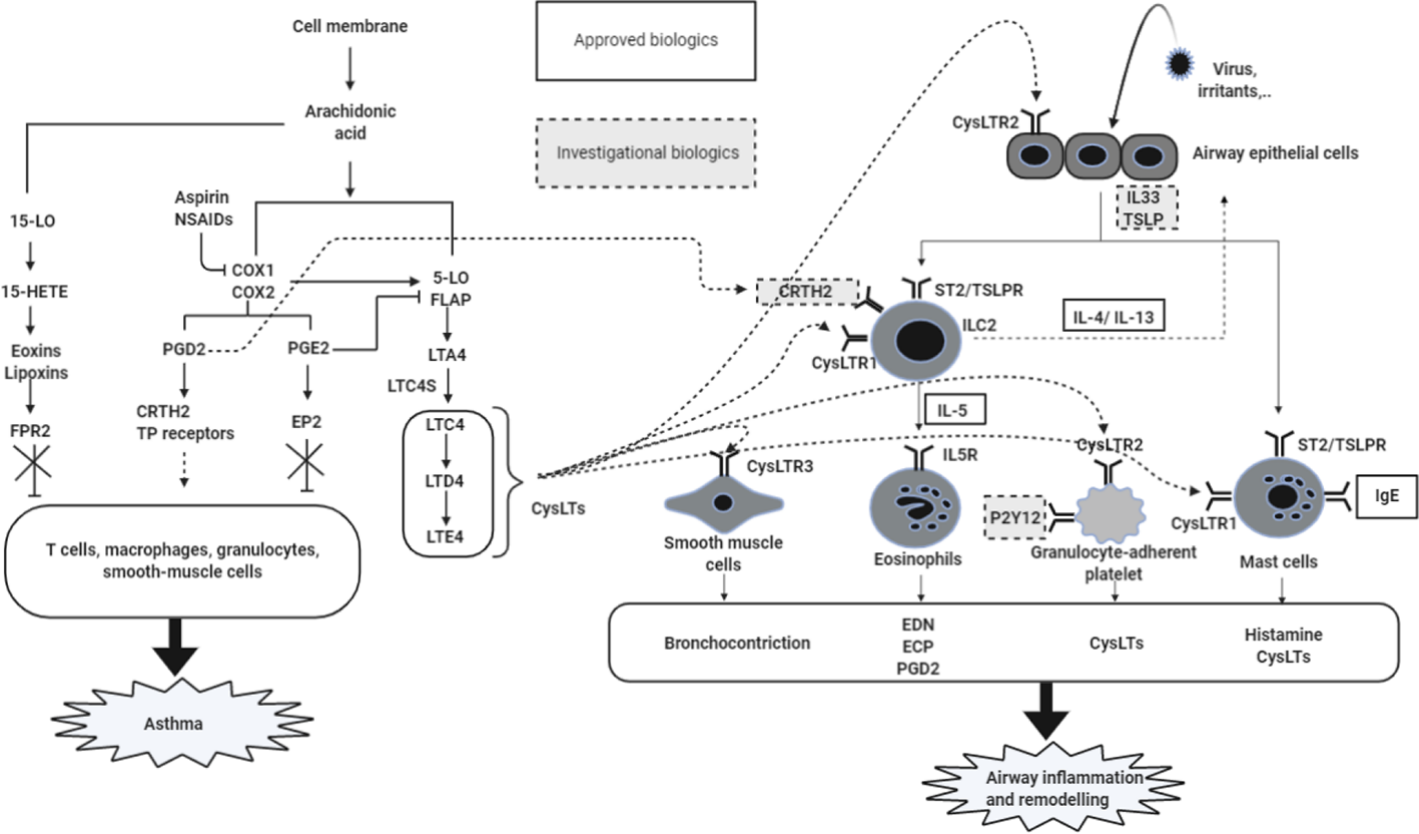
Mechanisms of airway inflammation in NERD. Increased levels of CysLTs and PGD_2_ as well as a decrease in the PGE_2_ level caused by the AA metabolism dysregulation are the main mechanism for promoting the severity of NERD. Released CysLTs, PGD_2_, and PGE_2_ regulate inflammatory cells *via* receptors expressed on individual cells (eosinophils, ILC2, mast cells, smooth muscle cells, granulocyte-adherent platelet, and neutrophils). These activated cells release cytokines, histamine, CysLTs, and PGD_2_, contributing to airway inflammation and remodeling in airway mucosa of NERD patients. 5-LO, 5-lipoxygenase; COX, cyclooxygenase; CysLTs, cysteinyl leukotrienes; PGs, prostaglandins; TBX, thromboxane; LT, leukotrienes; 15-HETE, 15-hydroeicosatetraenoic acid; FPR2, formyl peptide receptor 2; CysLTR, cysteinyl leukotrienes receptors; LTC4S, LTC4 synthase; EP2, E prostanoid 2; CRTH2, chemoattractant receptor-homologous molecule expressed on TH2 cells; TP receptors, T prostanoid receptors; IL, interleukin; TSLP, thymic stromal lymphopoietin; TSLPR, TSLP receptor; ILC2, innate lymphoid type 2 cells; Th2: T helper 2; ECP, eosinophil cationic protein; EDN: eosinophil-derived neurotoxin; IL5R, interleukin 5 receptor.

In the COX and LOX pathways, AA is metabolized to CysLTs (mostly LTE_4_, *via* 5-lipoxygenase [5-LO] and LTC_4_ synthase [LTC4S]), prostaglandin (PG) pathway (PGE_2_, PGF_2_, PGI_2_ and PGD_2_) and thromboxanes (TBX) A_2_ by PG synthase and TBX synthase (Szczeklik, 1990), where enhanced synthesis of CysLTs synthesis with reduced level of PGE_2_ is a major finding in NERD (Pham et al., 2016; Pham et al., 2017; Lee et al., 2018a; Yin et al., 2020). NERD patients have higher levels of CysLTs (especially LTE_4_) mainly derived from various inflammatory cells, including neutrophils, monocytes, and basophils, eosinophils and mast cells, which further increases after ASA/NSAID exposure compared to asthmatic patients with ASA/NSAID tolerance (ATA). Moreover, the increased expression of 5-LO and LTC4S was noted in NERD patients with overproduction of CysLTs; increased CysLTs bind to CysLT receptor 1/2, subsequently inducing bronchoconstriction and amplifying inflammatory signal pathways (Jonsson, 1998; Yonetomi et al., 2015; Steinke and Wilson, 2016; Sekioka et al., 2017). Among PGs, PGE_2_/PGD_2_ play a major role in the pathogenesis of NERD. Increased PGD_2_ (released from mast cells and eosinophils) binds to prostanoid receptors to induce bronchoconstriction (Säfholm et al., 2015), and also binds to chemoattractant receptor-homologous molecule expressed on TH2 cells (CRTH2) to induce chemotaxis and activate eosinophils/basophils/Th2 cells/innate lymphoid cells (ILC2) (Hirai et al., 2001; Woessner, 2017), accelerating type 2 airway inflammation (Chang et al., 2014). The down-regulation of PGE_2_ biosynthesis, especially in peripheral blood leukocytes, nasal epithelial cells and nasal fibroblasts, was noted in patients with NERD (Laidlaw and Boyce, 2013; Cahill et al., 2016; Pham et al., 2017). PGE_2_ has protective effects against bronchoconstriction, recruitment of eosinophils and degranulation of mast cells after binding to E prostanoid 2 (EP_2_) receptors (Feng et al., 2006; Sturm et al., 2008); therefore, reduced levels of PGE_2_ in NERD cannot suppress the signal of 5-LO pathways through IL-10-dependent mechanisms (Harizi et al., 2003). Furthermore, the lower expression of EP_2_ receptors is closely associated with abnormal regulation of the autocrine loop involved in COX pathways (IL-1R1, COX-2, mPGES) in NERD patients (Cahill et al., 2015; Machado-Carvalho et al., 2016). This can be explained that COX-2 could not sufficiently produce PGH_2_ (the first unstable precursors of PG products from AA metabolism) without COX-1 (Uematsu et al., 2002). Therefore, reduction in PGE_2_ and its receptor levels could contribute to CysLTs overproduction in NERD patients. Lipoxin (LX) A4 and its epimer (15-epi-LXA4) are also called as the ASA-triggered lipoxins, and have antiinflammatory effects in airway inflammation (Pham et al., 2017; Sokolowska et al., 2020). Their receptor termed formyl peptide receptor 2 (FPR_2_) is expressed on human neutrophils, eosinophils, macrophages, T cells, ILCs (ILC2 and NK cells) and epithelial cells of the respiratory tract. After binding their receptors, it leads to the restoration of epithelial barrier function and resolution of allergic inflammation through down-regulation of chemotaxis and cell activation (Barnig et al., 2013; Sokolowska et al., 2020). In the context of NERD, the concentration of LXA4 in the whole blood, sputum and bronchoalveolar lavage fluid, and 15-epi-LXA4 in the urine from NERD patients were lower than those in ATA patients. Additionally, their level has a negative correlation with worsening of airflow obstruction in patients with severe asthma (Christie et al., 1992; Sanak et al., 2000; Kupczyk et al., 2009; Yamaguchi et al., 2011). There was a significant increase in the FPR_2_ expression of NK cells and ILC2s from patients with severe asthma compared with those with milder asthma (Barnig et al., 2013). All of the studies suggested that LXA4 and its epimer can be considered the potential therapeutics in the treatment of NERD (**Figure 1**). NSAID-induced inhibition of the COX pathway leads to shunting of AA metabolism down the 5-LO arm (Palikhe et al., 2009; Dominas et al., 2020). This is indirectly evidenced through the decreased level of antiinflammatory PG/LX (LXA_4_, 15-epi-LXA_4_, PGE_2_) and increased levels of the pro-inflammatory CysLTs (Christie et al., 1992; Sanak et al., 2000; Harizi et al., 2003; Kupczyk et al., 2009; Yamaguchi et al., 2011).

### Enhanced Type 2 Airway Inflammation

NERD is characterized by persistent eosinophil activation (presenting severe asthma, CRS and NPs) and CysLTs overproduction in which increased CysLTs contributes to driving type 2 inflammatory responses (Lee et al., 2018a; Rusznak and Peebles, 2019; Taniguchi et al., 2019). The key inflammatory cells in NERD are eosinophils and mast cells, which are closely interacting with other inflammatory and structural cells including basophils, platelets, neutrophils and epithelial cells. Regarding the activation mechanisms of eosinophils, both Th2 *cells* and ILC2 could activate eosinophils *via* release of IL-4, IL-5 and IL-13; moreover, activated eosinophils release the eosinophil extracellular traps (EETs), enhancing type 2 inflammation *via* interacting with epithelial cells and autocrine functions of eosinophils in the asthmatic airway (Pham et al., 2017; Choi et al., 2019b; Yin et al., 2020). There have been some data demonstrating epithelial dysfunction related to type 2 inflammation in NERD: 1) lower levels of SPD (protective function against eosinophilia) (Choi et al., 2019a), 2) increased epithelial folliculin and periostin levels (Kim M. A. et al., 2014; Trinh et al., 2018; Choi et al., 2019b), 3) increased CysLT-induced signaling (binding to CysLT2R or CysLT3R) in airway epithelial cells to induce the release of pro-inflammatory cytokines including IL-33, TSLP and IL-25 (Corrigan et al., 2005), leading to type 2/eosinophilic inﬂammation and remodeling in NERD (Ulambayar et al., 2019).

Recent studies suggested that the activation of neutrophils may be related to the severity of airway inflammation in NERD (Kim et al., 2019), although the exact mechanism is still not fully elucidated. Increased LTB4 levels (mostly formed from neutrophils) and reactive oxygen species release after N-formyl-methionyl-leucyl-phenylalanine stimulation were noted in patients with NERD compared to ATA patients (Mita et al., 2004; Kim et al., 2019). In addition, platelets are activated by CysLTR2 on their surfaces to release IL33 and to interact with leukocytes through binding P-selectin (CD62P)–P-selectin glycoprotein ligand 1, GPIIb/IIIa-Mac-1 and CD40 ligand (CD40L)–CD40 (Laidlaw et al., 2012; Mitsui et al., 2016; Liu et al., 2019; Taniguchi et al., 2019). The activation of platelets and adherent leukocytes with platelets leads to the transmigration of leukocytes into inflammatory airway tissue with increased CysLTs, suggesting that platelet-aggregated granulocytes promote severe and persistent airway inflammation in NERD patients (Laidlaw and Boyce, 2013; Laidlaw et al., 2014; Mitsui et al., 2016).

### Genetic Mechanisms

Many genetic studies have focused on CysLTs-related and eosinophil activating genes (major pathogenic mechanisms) according to single nucleotide polymorphisms (SNPs) and genome-wide association studies (GWASs) (Pavón-Romero et al., 2017). (**Table 1**) *HLA* DPB1*0301 has been regarded as a strong genetic marker and replicated in the 2 ethnic groups Polish and Korean populations (Dekker et al., 1997; Choi et al., 2004a). Patients suffering from this allele manifested the typical clinical characteristics of NERD, and had lower FEV1 levels and a higher prevalence of CRS and/or NPs (Choi et al., 2004a). The GWAS demonstrated several significant SNPs (*HLA-DPB1*, rs3128965, *DPP10* rs17048175 in a Korean population, *TSLP* rs1837253 in a Japanese population, etc.) which were associated with the phenotypes of NERD (Park et al., 2013; Kim S. H. et al., 2014; Kim et al., 2015). The genetic polymorphism studies identifying the SNPs related to CysLTs synthesis demonstrated several significant SNPs: the promoter polymorphisms at the *LTC4S* -444 A>C in a Polish population (Sanak et al., 1997), although it was not replicated in the other populations as the US, Japanese and Korean (Van Sambeek et al., 2000; Kawagishi et al., 2002; Choi et al., 2004b). The SNPs of G-coupled receptors (*CysLTR1* -634C>T, -475 A>C, -336 A>G, *CysLTR2* -819 T>G, 2078 C>T, 2534 A>G) lead to amplify the biological activity of CysLTs, the SNPs of prostanoid receptor genes (*PTGER2* -616 C>G, -166 G>A, *PTGER3* -1709 T>A, *PTGER4* -1254 A>G, *PTGIR* 1915 T>A, *TBXA2R* -4684 C>T, 795 T>C) were associated with the development of NERD (Park et al., 2005; Kim et al., 2006; Kim et al., 2007). Regarding the SNPs related to eosinophil activation, including those of the chemokine CC motif receptor (*CCR3* −520 T>C), chemoattractant receptor molecular expressed in Th2 cells (*CRTH2* −466 T>C) and *IL5R* (-5993 G>A), were reported (Kim et al., 2008; Palikhe et al., 2010; Losol et al., 2013). Epigenetic factors, including exposure to NSAIDs and other stimuli, be also revealed to contribute to the development of NERD (Pham et al., 2017; Yin et al., 2020); DNA methylation associated with some SNPs (PGE synthesis, PGS, ALOX4AP, LTC4S, etc.) may contribute to presenting more severe phenotypes of NERD (Lee et al., 2019). Further replication studies in diverse ethnic groups are needed to clarify their functional roles in parallel with other omics markers with subphenotype classification.

**Table 1:** Genetic polymorphisms associated with NERD.

	Gene	SNP	Analysis methods	Ethnic group	Patients	OR (95% CI)	*P*-value (compared with ATA)	Reference
CysLTs overexpression	*LTC4S*	−444 A>C	Amplified-fragment single-strand conformation polymorphism	Polish	NERD: 47, ATA: 64, NC: 42	3.89 (1.57–8.98)	<0.001	(Sanak et al., 1997)
	*CysLTR1*	−634 C>T, −475 A<C, −336 A<G	Direct sequencing method	Korean	NERD: 105, ATA: 110, NC: 125	2.71 (1.10–6.68) 2.89 (1.14–7.28)	0.020	(Kim et al., 2006)
	*CysLTR2*	−819 T>G 2,078 C>T 2,534 A>G	ABI PRISM 3700 DNA analyzer	Korean	NERD: 134, ATA: 66, NC: 152	2.04 (1.06–3.85) 2.28 (1.19–4.40) 2.02 (1.07–3.84)	0.031 0.013 0.031	(Park et al., 2005)
	*PTGER2*	−616 C>G −166 G>A	Direct sequencing	Korean	NERD: 108, ATA: 93, NC: 140	0.64 (0.42–0.98) 2.60 (1.14–5.92)	0.038 0.023	(Kim et al., 2007)
	*PTGER3*	−1,709 T>A				3.02 (1.04–8.80)	0.043	
	*PTGER4*	−1,254 A>G				1.77 (1.08–2.90)	0.024	
	*PTGIR*	1,915 T>A				0.41 (0.20–0.86)	0.018	
	*TBXA2R*	−4,684 C>T				0.42 (0.19–0.91)	0.032	
		795 T>C				0.67 (0.45–1.00) 2.57 (1.09–6.09)	0.049 0.032	
Enhancement of type 2 inflammation	*CCR3*	−520 T>C	MDR method	Korean	NERD: 94, ATA: 152	ND	ND	(Kim et al., 2008)
	*CRTH2*	−466 T>C	Primer extension methods	Korean	NERD: 107, ATA: 115, NC: 133	ND	0.044 (TT) 0.037 (CC)	(Palikhe et al., 2010)
	*IL5R*	−5,993 G>A	Primer extension method	Korean	NERD: 139, ATA: 171, NC: 160	ND	0.685 (GG) 0.495 (AG) 0.408 (AA)	(Losol et al., 2013)
Others	*HLA*	DPB1*0301	DNA methods	Polish	NERD: 59, ATA: 57, NC: 48	5.3 (1.90–14.40)	<0.001	(Dekker et al., 1997)
			ABI 3100 Genetic analyzer	Korean	NERD: 76, ATA: 73, NC: 91	5.2 (1.80–14.70)	0.004	(Choi et al., 2004a)
	*HLA-DPB1*	rs3128965	Affymetrix Genome-Wide Human SNP array	Korean	NERD: 264, ATA: 387, NC: 238	1.8 (1.22–2.68) 3.1 (094–10.70)	0.098 (AG) 0.001 (AA)	(Kim S. H. et al., 2014)
	*HLA-DPB1*	rs104215	GoldenGate assay with the VeraCode microbead	Korean	NERD: 117, ATA: 685	2.40 (1.68–3.42)	<0.001 (fine-mapping study)	(Park et al., 2013)
	*DPP10*	rs17048175	Affymetrix Genome-Wide Human SNP array	Korean	NERD: 139, ATA: 171, NC: 160	ND	0.083 (TT) 0.072 (CT) 0.022 (CC)	(Kim et al., 2015)

NERD, NSAID-exacerbated respiratory disease; ATA, aspirin-tolerant asthma; CysLTR, cysteinyl leukotriene receptor; LT, leukotriene; PG, prostaglandin; TX, thromboxane; CRTH2, chemoattractant receptor homolog expressed by type 2 helper T cells; CCR, chemokine receptor; HLA, human leukocyte antigen; DPP, dipeptidyl peptidase; IL, interleukin; ND, no data.

## Diagnosis

A diagnosis of NERD is fundamentally based on the patient’s history. NERD is suspected in patients having a history of upper/lower respiratory reactions after ingestion of ASA/NSAIDs or suffering from asthma along with CRS and NPs, (Choi et al., 2015). Some patients have a definitive history of adverse reactions to ASA/NSAIDs: however, many patients have not experienced hypersensitivity reactions (Palikhe et al., 2009). One study showed that 14% of patients who thought they had NERD based on symptoms were negative for oral aspirin challenge (Dursun et al., 2008). Thus, ASA challenge, as the gold standard for diagnosing NERD, is required to confirm or exclude hypersensitivity in patients with unclear history of adverse reactions.

There are 3 types of the ASA challenge test *via* the oral, bronchial and nasal routes. The oral challenge test is a more commonly used and convenient approach compared to other challenge tests in that it mimics natural exposure (Adkinson et al., 2013). It may be more suitable for investigating systemic adverse reactions to NSAIDs. Bronchial challenge with lysine-aspirin is safer and quicker, but shows lower sensitivity than the oral test. Nasal challenge is recommended for patients with predominant nasal symptoms, but the sensitivity is lower (Lee et al., 2018a; Kowalski et al., 2019). The EAACI recommended the oral challenge protocol with starting 20-40 mg of aspirin and gradually increasing the dose at 2 hour intervals. When no reactions occur within 3 hours after 325 mg of aspirin, the challenge is considered to be negative (Kowalski et al., 2019). Patients with lower FEV1 (<70% of the predicted value) or unstable asthma status are not recommended, and the test should be performed in a hospital with resuscitative equipment under the supervision of special training physicians (Adkinson et al., 2013). These tests may be influenced by bronchial hypersensitivity, ASA dosage, and the concurrent use of leukotriene modifier drugs and antihistamines (White et al., 2005; White et al., 2006). When patients are false-negative for ASA challenge, subsequent confirmatory challenges are recommended for holding leukotriene modifier drugs, antihistamines and oral corticosteroids for at least 1 week and employing high-dose ASA challenges (White et al., 2013).

There is no *in vitro* test available for the diagnosis of NERD. LTE_4_ (especially in urine) is suggested to be the most reliable biomarker for the diagnosis of NERD. Several studies demonstrated that patients with NERD had higher baseline concentrations of urinary LTE_4_ as well as greater increase after aspirin/NSAID exposure than in patients with ATA, suggesting that urine LTE_4_ level could be used as a clinical diagnostic test (Hagan et al., 2017; Bochenek et al., 2018). Recent studies demonstrated higher levels of serum periostin, and folliculin as potential biomarkers of NERD, however, further validation studies are needed in other cohorts (Kim M. A. et al., 2014; Trinh et al., 2018). The Polish group proposed the Aspirin-Sensitive Patients Identification Test (ASPI Test), however, it was not replicated in other centers (Kowalski et al., 2005). Despite the basophil activation test (BAT) has been investigated for *in vitro* diagnosis of NERD, variable values of sensitivity and specificity were reported depending on the protocols used, remaining limitations of the clinical use (Schafer and Maune, 2012). More efforts are needed to establish *in vitro* diagnostic tests for reducing the risks of challenge tests with identifying reliable biomarkers for the diagnosis of NERD and the classification of its subphenotypes.

## Management

The standard management of NERD involves the guidelines established for the management of asthma and CRS with ASA/NSAID avoidance. The complete avoidance of culprit agents and cross-reacting NSAIDs with use of alternative agents (highly selective COX-2 inhibitors such as celecoxib, and partial inhibitors such as acetaminophen, meloxicam or nimesulide) is essential. ASA desensitization can be beneficial for NERD patients when indicated.

### Pharmacologic Treatment

Treatment strategies for asthma should follow stepwise management guidelines with maintaining inhaled corticosteroids with or without long-acting beta 2 agonists, leukotriene modifier drugs and/or biologic agents on the basis of disease severity and rescue medications (GINA-guideline, 2020). Because the overproduction of CysLTs is a key feature in the pathogenic mechanisms, targeting the leukotriene pathway with CysLT1 receptor antagonists (montelukast, zafirlukast and pranlukast) and 5-LO inhibitors (zileuton) should be considered to improve upper and lower airway symptoms. Several studies have shown that these leukotriene modifiers lead to improvement in asthma symptoms, pulmonary function, quality of life, nasal function and lower use of bronchodilators (Rodriguez-Jimenez et al., 2018).

Initial treatment for CRS includes intranasal corticosteroids with intranasal saline irrigation. Intranasal corticosteroids have shown to be highly effective in reducing nasal inflammation and in shrinking NPs, which are recommended as a first-line treatment in patients with CRSwNP (Choi et al., 2015; Simon et al., 2015; Rodriguez-Jimenez et al., 2018). Because rinsing the nasal cavities with saline is helpful in removing secretions and washing away allergens and irritants, nasal irrigation prior to administration of topical medications can improve the response to the medications (Simon et al., 2015; Rodriguez-Jimenez et al., 2018). Systemic corticosteroids and broad-spectrum antibiotics can be additionally required according to the severity of nasal symptoms. Adding antihistamines or oral/nasal decongestants may provide symptom relief (Adkinson et al., 2013).

Despite the heterogeneity of NERD, therapeutic approaches have been proposed according to symptom severity. However, these different phenotypes contribute to the variability in response to treatment. A recent study found that clinical severity and courses differ among the 4 subtypes of NERD, which affect antiasthmatic medications required (Lee et al., 2017). Subtype 1/2 patients had severe clinical courses, requiring higher-dose of antiasthmatic medications including higher dose of ICS and systemic corticosteroids, while subtype 3 patients required low doses of these drugs with less frequent asthma exacerbation. These results suggest that a personalized approach according to subtype classification is needed to achieve better outcomes in the management of NERD.

### ASA Desensitization

ASA desensitization is an effective treatment option when standard medical treatments are not effective or daily ASA/NSAIDs therapy is required for other medical conditions, such as coronary artery disease or chronic inflammatory disease (Stevenson and Simon, 2006). Multiple studies have demonstrated the effectiveness of ASA desensitization in reducing NP size and the need for sinus surgery as well as in improving nasal and bronchial symptoms with decrease in the doses of topical and oral corticosteroids (Swierczynska-Krepa et al., 2014; Waldram et al., 2018). A recent study showed the long-term safety and efficacy of ASA desensitization in patients who underwent continuous daily ASA therapy for more than 10 years (Walters et al., 2018). ASA desensitization is a provocative procedure by starting at low doses of ASA and gradually increasing to the dose of 650 to 1300 mg over a period of 1 to 3 days, which can induce hypersensitivity reactions (White and Stevenson, 2018). Thus, as safety is an important issue, ASA desensitization should be carried out in a well-equipped hospital under the supervision of special training physicians. The protocol with gradually increasing the dose over 2 days was suggested by the EAACI to secure safety and efficacy of aspirin desensitization (Kowalski et al., 2019).

### Biologics

The emergence of biologics in the management of asthma and CRSwNP has represented potential and promising therapy for NERD. New biologics targeting type 2 cytokines, such as IL-4, IL-5 and IL-13 as well as IgE, have been reported in clinical trials, which could reduce asthma exacerbation and oral corticosteroid use, and improve lung function (Kim and Jee, 2018; McGregor et al., 2019). In addition, they have been shown to improve nasal symptom severity and reduce NP size in patients with CRSwNP, leading to a significant increase in quality of life (Bachert et al., 2020). Because NERD is strongly associated with mast cell activation and eosinophilic airway inflammation, the efficacy of biologics may be different from those usually observed in severe asthma (Hayashi et al., 2016). Here, we summarized the available studies for these biologics in patients with NERD (**Table 2**).

**Table 2:** Biologics in NERD patients: Summary of available studies.

Biologics(Target)	Study design(Number of participants)	Route, Dose and Study period	Efficacy outcomes	Reference
Omalizumab (IgE)	Double-blind, randomized, placebo-controlled trial (16 Omalizumab vs. 16 Placebo)	Subcutaneous injection every 2 or 4 weeks based on total IgE level and body weight for 3 months	Improvement in ACT, ACQ-6, SNOT-22 and VAS scores in omalizumab group compared with placebo group after 3-month treatment. (All, *P*<.001) Improvement in FEV1 (%) in omalizumab group compared with placebo group after 3-month treatment (*P*=.003)	(Hayashi et al., 2020)
Omalizumab (IgE)	Retrospective analysis (29 Omalizumab)	Subcutaneous injection for 1 year	Reduction in use of OCS and SABA during 1 year on omalizumab treatment compared with 1 year before initiating omalizumab. (All, *P*=.001)	(Jean et al., 2019)
Dupilumab (IL-4Rα)	*Post hoc* analysis (8 Dupilumab vs. 11 Placebo)	Subcutaneous injection of 300 mg weekly for 16 weeks	Improvement in NPS, ACQ-5 and SNOT-22 total scores in dupilumab group compared with placebo group after 16-week treatment (All, *P*<.005) Changes in FEV1 (L) from baseline in dupilumab group compared with placebo group after 16-week treatment. (*P*<.05)	(Laidlaw et al., 2019)
Mepolizumab (IL-5)	Retrospective analysis (14 Mepolizumab)	Subcutaneous injection of 100 mg every 4 weeks for 3 months	Reduction in absolute eosinophil count from baseline after 3-month treatment. (*P*=.001) Improvement in SNOT-22 and ACT scores from baseline after 3-month treatment. (*P*=.005 and *P*=.002, respectively) No significant improvement in FEV1 (%) from baseline (*P*=.16)	(Tuttle et al., 2018)
Reslizumab (IL-5)	*Post hoc* analysis (28 Reslizumab vs. 28 Placebo)	Intravenous injection of 3 mg/kg every 4 weeks for 52 weeks	Difference in frequency of asthma exacerbation in reslizumab, 0.29 vs placebo, 1.95 (*P*=.001) during 52-week treatment. Changes in FEV1 (L) from baseline in reslizumab, 0.327L vs placebo, 0.002L (P<.001) after 52-week treatment.	(Weinstein et al., 2019)

IL, interleukin; IL-4Rα, interleukin-4 receptor alpha subunit; ACT, asthma control test; ACQ-6, 6-item asthma control questionnaire; SNOT-22, 22-item sino-nasal outcome Test; VAS, visual analog scale; OCS, oral corticosteroid; SABA, short-acting β2 agonist; NPS, nasal polyp score; ACQ-5, 5-item asthma control questionnaire.

Omalizumab, a humanized recombinant monoclonal anti-IgE antibody, prevents IgE from binding to its high-affinity receptor and reduces Fc receptor expression on mast cells and basophils, subsequently suppressing their activation (Chang et al., 2015). Several studies have suggested the efficacy of omalizumab in the management of NERD, demonstrating a reduction in asthma exacerbation and the need for systemic steroids and short acting beta-2 agonist (SABA) as well as an improvement in upper and lower airway symptoms (Hayashi et al., 2016; (Lee et al., 2018b; Jean et al., 2019). Furthermore, there are some studies suggesting that omalizumab treatment can be beneficial for reducing respiratory symptoms during ASA desensitization and even can restore ASA tolerance without the need for ASA desensitization (Phillips-Angles et al., 2017; Lang et al., 2018; Hayashi et al., 2020). Omalizumab could improve upper and lower airway symptoms with suppression in urinary markers of mast cell activation, LTE_4_ and PGD_2_ metabolites, in patients with NERD and lead to the development of ASA tolerance with a reduction in urinary LTE_4_ concentrations during oral ASA challenge (Hayashi et al., 2016; Hayashi et al., 2020), suggesting that omalizumab has inhibitory effects on mast cell activation in NERD.

Dupilumab is a human monoclonal antibody that targets the IL-4α receptor and inhibits signaling of both IL-4 and IL-13. Although the study was conducted in a small number of patients with NERD, dupilumab could improve nasal and asthma-related symptom scores and lung functions (Laidlaw et al., 2019), although studies with a larger sample size are needed to confirm its effectiveness. Mepolizumab and reslizumab are both monoclonal antibodies that prevent IL-5 from binding to its receptor on eosinophils, and benralizumab is a monoclonal antibody that targets the alpha subunit of the IL-5 receptor. The respiratory tract of NERD patients is characterized by intense eosinophilic inflammation, with higher levels of eosinophils in NPs and bronchial mucosa biopsies than in ATA patients (Tuttle et al., 2018; Eid et al., 2020). These biologics inhibiting IL-5, eosinophilic maturation and differentiation factor could be effective in the management of patients with NERD (Choi et al., 2004b). In addition, based on recent study results on the pathogenic mechanisms, P2Y12 receptor antagonists, CRTH2 antagonists and anti-TSLP/IL-33 antibodies could be potential options in the management of NERD patients (Rodriguez-Jimenez et al., 2018).

Considering the heterogeneity of NERD phenotypes/endotypes, selecting right patients and right targets (biologics) are essential in the management of NERD. In phenotypic clusters of NERD, subtype 4 patients (NERD with urticaria) would need omalizumab as an effective option, which can inhibit activated basophils and mast cells, the key elements of NERD and urticaria (Lee et al., 2017); subtype 2 patients with severe eosinophilia may need anti-IL-5 as a first option. Despite the development of biologic therapies, unmet needs remain in NERD patients to be understood with regard to their comparative efficacy and long-term safety. Further studies are needed to answer questions on the selection of right patients and targets with right safety.

### Dietary Interventions

Dietary intervention may be beneficial for controlling symptoms in patients with NERD. Some studies demonstrated that restricting dietary salicylates, including fruits, vegetables, berries, herbs, and spices, improves nasal and asthmatic symptoms, which can be explained by the known contribution of salicylates in the pathogenesis (Ta and White, 2015; Sommer et al., 2016). A previous study showed that alcohol ingestion can more commonly lead to upper and lower respiratory reactions in NERD patients, although the underlying mechanism is not clear (Cardet et al., 2014). Thus, restricting the diet, when experienced respiratory symptoms after the ingestion, can be additionally effective.

## Conclusion

Patients with NERD present with a variety of clinical features affected by chronic type 2 eosinophilic inflammation with the overproduction of CysLTs in the upper and lower airways. Although NERD tend to be associated with severe asthma and CRSwNP, an improved understanding of clinical features and underlying pathogenesis of NERD will aid in diagnostic evaluations and new therapeutic strategies for improving clinical outcomes. With the increasing recognition of phenotypic heterogeneity of NERD, efforts are needed to establish precision medicine strategies tailored to individual phenotypes/endotypes with potential biomarkers.

## Author Contributions

The clinical features, diagnosis and treatment of NERD were described by S-DW and the pathophysiologic mechanisms including molecular genetic mechanisms were described by QL. This article was written under supervision of H-SP. She, as corresponding author, performed the overall design and review of this article.

## Funding

This study was supported by a grant of the Korea Health Technology R&D Project through the Korea Health Industry Development Institute, funded by the Ministry of Health & Welfare, Republic of Korea (H116C0992).

## Conflict of Interest

The authors declare that the research was conducted in the absence of any commercial or financial relationships that could be construed as a potential conflict of interest.
